# Hematologic manifestations of coronavirus disease 2019 in children: Case-series report and a review

**DOI:** 10.3389/fped.2022.935236

**Published:** 2022-08-16

**Authors:** Grace Onimoe, Juan Alvarado, Anita Boakye

**Affiliations:** ^1^The MetroHealth System, Cleveland, OH, United States; ^2^Department of Pediatrics, MetroHealth Medical Center, Case Western Reserve University, Cleveland, OH, United States

**Keywords:** COVID-19, children, hematologic, manifestations, case series

## Abstract

On 11 March 2020, coronavirus disease 2019 (COVID-19) caused by severe acute respiratory syndrome coronavirus (SARS-CoV-2) was declared as a pandemic by the World Health Organization (WHO). As the COVID-19 pandemic has ravaged worldwide, children have not been unaffected. Information gleaned from adult experience with the disease has aided in disease detection and treatment strategies in children. Numerous cases have been described in adult literature about hematologic manifestations of COVID-19. This case series aims to report several hematologic presentations in patients with COVID-19 and multisystem inflammatory syndrome in children (MIS-C, an immune-mediated reaction leading to severe COVID-19 illness) with and without a primary hematologic disorder.

## Introduction

Coronavirus disease 2019 (COVID-19), caused by severe acute respiratory syndrome coronavirus (SARS-CoV-2), was announced as a pandemic by the World Health Organization (WHO) on 11 March 2020. Since then, the COVID-19 pandemic has significantly impacted healthcare systems, socioeconomic aspects, and livelihoods (1–5). COVID-19 has had overwhelming morbidity and mortality in the adult population. However, the severity of COVID-19 in the pediatric population has also been reported (6, 7). COVID-19, though primarily respiratory in origin, results in a multisystemic disorder that involves the cardiovascular, respiratory, gastrointestinal (GI), neurologic, hematopoietic, and immunologic systems (8). Of particular interest in this case series are the hematopoietic sequelae of COVID-19, which manifest themselves as laboratory abnormalities and coagulopathic and thrombotic events. Hematologic changes occurring early in COVID-19 include lymphocytopenia and thrombocytopenia, while the late-stage changes include more severe thrombocytopenia, neutrophil elevation, and severe coagulation disorder (9). We reported several pediatric case presentations and detailed hematologic findings.

## Case description and diagnostic assessments

### Case 1: Multisystem inflammatory syndrome in children with severe refractory thrombocytopenia

An otherwise healthy 4-year-old girl with a history of SARS-CoV-2 infection for 4 weeks before the presentation was admitted, followed by 4 days of sore throat, fatigue, and decreased oral intake. They had tested positive for COVID-19 on December 9, 2021 (2 months before presentation) and were symptomatic for approximately 1 week while quarantined at home. The patient was admitted to the pediatric floor with a suspected diagnosis of COVID-19 or multisystem inflammatory syndrome in children (MIS-C). The rheumatology service was then consulted. Initial laboratory results showed mild anemia (Hb, 10.9 mg/dl), neutrophilia (75%), lymphopenia (11%), pandemic (12%), elevated inflammatory markers (erythrocyte sedimentation rate (ESR), 100 mm/h; C-reactive protein (CRP), 23.6 mg/dl; ferritin, 350 ng/ml; procalcitonin, 46.25 ng/ml), hyponatremia (135 mmol/L), markedly elevated D-dimer (1,398 ng/ml), elevated B-type Natriuretic peptide test (BNP) (179 pg/ml), normal troponin I < 0.030 ng/ml, transaminitis (alanine aminotransferase (ALT), 57 IU/L and AST, 97 IU/L), and normal electrocardiogram (EKG). Due to suspected MIS-C, rheumatology recommended two doses of 1 g/kg intravenous immunoglobulin (IVIG) at 24 h interval. The patient later developed hypotension and persistent tachycardia refractory to resuscitation with crystalloid; she was started on 30 mg/kg/day methylprednisolone and transferred to the Pediatric Intensive Care Unit.

The patient required treatment with vasopressors due to worsening hypotension. Prophylactic anticoagulation with Lovenox was initiated. The hospital course was further complicated by acute hypoxic respiratory failure requiring intubation. Clinical evaluation supported a diagnosis of MISC-C as per CDC guidelines. Inflammatory markers were markedly elevated, necessitating treatment with anakinra at 2 mg/kg, and later increased to a maximum dose of 6 mg/kg. Due to abdominal distension and firmness to palpation, ultrasound (US) and CT scans were obtained and showed signs concerning acalculous cholecystitis. Severe third spacing, electrolyte imbalances (potassium, phosphorus, and sodium), and hypoalbuminemia occurred. Gastroenterology and surgery teams evaluated the patient and recommended a conservative approach (non-surgical approach). Electrolyte replacements and albumin infusions were administered. Echocardiograms obtained showed relatively normal cardiac muscle function. In the following days, a complete blood count (CBC) revealed a significant drop in hemoglobin (nadir of 6.6 mg/dl), which required blood transfusions on two separate occasions, and severe thrombocytopenia (bottom of 23 K/μl). Workup for thrombotic thrombocytopenic purpura and disseminated intravascular coagulation was negative. Literature search assured that this was post-MIS-C immune thrombocytopenia observed while patients are recovering from MIC-S, and treatments known for treating ITP are sufficient. Two doses of IVIG were given, after which platelets increased slightly; subsequently, two doses of romiplostim were administered, after which platelet count increased substantially. As the patient improved clinically, inflammatory markers down trended, and lymphopenia and thrombocytopenia resolved. The patient was transferred to the Pediatric Ward for methylprednisolone, anakinra wean, physical therapy, and nutritional optimization. She was discharged home on hospital day 21.

### Case 2: Sickle cell disease with fever and vaso-occlusive pain crises

A 15-year-old girl with a history of sickle cell disease (SCD)-HbSC genotype, on hydroxyurea and voxelotor, presented to the Emergency Department (ED) with a 3-day history of fever, chest, abdomen, and lower extremity pain that failed to respond to pain medications. On presentation to the ED, she was afebrile and stable on room air (RA) without signs of respiratory distress. Initial laboratory results showed hemoglobin of 9.8 g/dl (baseline, 11 g/dl), lymphopenia, and elevated reticulocyte count to 3.5%; SARS-CoV-2 PCR was positive, and chest X-ray was negative for acute chest syndrome. She was admitted to the inpatient unit to manage a vaso-occlusive pain crisis. Pain control was started with a hydromorphone patient-controlled analgesia (PCA) pump and scheduled IV ketorolac. She remained afebrile and stable on RA. Her pain was adequately controlled, which allowed her to later transition to oral oxycodone and ibuprofen. She was discharged home on day 3 of hospitalization.

### Case 3: Sickle cell disease with fever and vaso-occlusive pain crises

A 16-month-old boy with a history of SCD-HbSC genotype, on twice-daily amoxicillin prophylaxis, presented to the ED with 4 days of cough, vomiting, one day of fever, and questionable limping. He was afebrile and stable on RA without signs of respiratory distress. Initial laboratory results showed hemoglobin of 10.6 g/dl (baseline, 11.5 g/dl), elevated reticulocyte count to 3%, and average differential count; SARS-CoV-2 PCR was positive, and chest X-ray was negative for acute chest syndrome. Liver function tests and basic metabolic panel were within the normal ranges. He was admitted to the Pediatric Ward to manage vaso-occlusive pain crisis and rule out sepsis. The pain was adequately controlled with morphine and acetaminophen. He remained afebrile and stable on RA; blood culture showed no growth for the past 48 h. The pain was adequately controlled, which allowed the transition to oral oxycodone and ibuprofen. Hemoglobin remained stable with a decrease in reticulocyte count. He was discharged home on day 3 of hospitalization.

### Case 4: Hereditary spherocytosis with acute on the chronic hemolysis

A 12-year-old boy with a history of hereditary spherocytosis and previously vaccinated against SARS-CoV-2 presented to Emergency Care with a 2-day-history of cough, congestion, abdominal pain, nausea, and vomiting. On examination, he was afebrile, stable on RA, and had significant icteric sclerae without hepato-splenomegaly or abdominal tenderness. Laboratory results showed slight decrease in red blood cell count (3.66 M/μl vs. 3.81 M/μl), hemoglobin (11 g/dl vs. 10.8 g/dl), hematocrit (29.3% vs. 29.7%), and mean corpuscular hemoglobin concentration (36.7 g/dl vs. 37 g/dl) compared to his baseline. Liver function tests showed elevated total bilirubin (3.2 mg/dl vs. 2.1 mg/dl) compared to baseline; gamma-glutamyl transpeptidase and lipase were within normal ranges, as CRP and ESR. Given the patient clinical stability, he was discharged home and under close monitoring for ongoing hemolysis. A repeat blood count obtained 48 h later showed a further drop in hemoglobin counts at 8.6 g/dl, and reticulocyte count was elevated at 13.6%. Hemoglobin returned to a baseline count of 11.3 g/dl after 1 week.

### Case 5: Post-coronavirus disease 2019 immune thrombocytopenia

A 13-month-old boy with a recent history of COVID-19 infection (SARS-CoV-2 PCR positive) 2 weeks before presented to Emergency Care for worsening petechiae over a 3-day course. The petechiae started on his stomach and chest, spreading to his extremities later. There were no previous episodes of mucosal bleeding or epistaxis, although parents described multiple bruises on the legs, some of which were attributed to bumps or falls. The initial laboratory results showed a platelet count of 10 K/μl, few burr cells on the peripheral smear, mild elevation of coagulation tests (PTT 44 s, PT 13.5 s, INR 1.22), normal fibrinogen (258 mg/dl), elevated Factor V (289%), slightly low Factor VII (48%) and normal Factor X (113%), elevated D-dimer (685 ng/ml), and normal inflammatory markers (ESR, 16 mm/h; CRP < 0.5 mg/dl; ferritin, 37.3 ng/ml). He was admitted to the inpatient unit for the management of thrombocytopenia, likely secondary to the recent COVID-19 illness. Treatment was initiated with 1 g/kg IVIG and 1 mg/kg methylprednisolone. Repeat CBC showed an increase of platelet count initially to 14 and 40 K/μl at 20- and 35-h post-treatment, respectively. He was discharged home and repeat platelet count checks achieved normal ranges within several weeks of hospital discharge, and platelet counts have remained stable.

## Discussion

Entry of the SARS-CoV-2 to human host cells requires the cellular receptor angiotensin-converting enzyme 2 (ACE) and serine protease TMPRSS2 for spike protein priming. The virus enters the target cells *via* interaction between viral surface S spike protein and ACE2 (type 1 integral membrane receptor) expressed in multiple tissues, i.e., lung, heart, kidney, GI, and vasculature (6, 10). Cleavage of the viral S protein is done by TMPRSS2, following which the viral RNA genome is released, and the viral replication cycle starts (6). After entry, the virus can multiply and disseminate into the airway after shutting down the IFN type 1 antiviral pathway and spreading from the lung to other ACE2-expressing tissues (6). The direct cytotoxic effect of the virus on endothelial cells and microvascular occur, followed by inflammation and excessive release of cytokines which further aids the development of a prothrombotic state (6).

Coronavirus disease 2019 is primarily a respiratory illness that ranges from asymptomatic infection to a more severe form of the disease that is multisystemic. Most children who develop COVID-19 have mild symptoms or remain asymptomatic. Numerous studies have reported children with certain underlying medical conditions, such as chronic respiratory illness, moderate-to-severe asthma, obesity, diabetes, sickle cell disease, and cancer (11). COVID-19 causes a variety of hematologic findings that have been reported in the literature. Changes in peripheral blood count, including lymphopenia, thrombocytopenia, and elevated D-dimer levels, are among the most reported results (12). ACE is expressed on lymphocytes, and the virus utilizes this to cause a direct cytotoxic effect leading to lymphopenia, ACE2 receptors in hematopoietic stem cells in BM-pancytopenia (depletes all forms of blood cells), and finally, TNF-alpha induction during the cytokine storm mediates cell apoptosis (13). Neutrophilia is preceded by a cytokine storm, superimposed bacterial infection (13). Thrombocytopenia results from the destruction of hematopoietic precursors in the marrow, destruction by virus-induced autoantibodies which form immune complexes and are cleared from the body, the consumption of platelets during the coagulation cascade, and thrombi formation (13).

The standard CBC changes from COVID reported in children include lymphopenia, leukocytosis/leukopenia, thrombocytopenia, and neutrophilia (14).

Other significant lab findings include elevation in inflammatory markers, i.e., CRP, ESR, and procalcitonin (14). A study pooled 24 studies involving 624 pediatric cases with lab-confirmed COVID-19, reporting data on 27 biomarkers (0–17.5 years, female 43%) (15). Only 6 of the 24 studies addressed lab findings in patients with severe COVID-19 illness. Due to limited data and high variation across these studies, it could not be analytically pooled. Observations revealed that a few patients had elevated WBC, and increased and decreased lymphocyte counts were observed at an equal frequency (18.4%). In addition, elevated D-dimer and PT could be seen (15). Lymphopenia, thrombocytopenia, and neutrophilia have all been used as hematologic biomarkers for prognosticating disease severity in adults (1). However, there are subtle differences in the pediatric population as lymphopenia is not observed as frequently in the pediatric population compared to the adult population, although the presence of lymphopenia can correlate with disease severity in the pediatric population (16). According to Per Cui et al., the reduction of lymphocytes in children was only 16%, but in adults, it was up to 43.1, 57.4, and 56%, respectively, reported by a previous meta-analysis; the reason for these differences may be related to the immune response of different organisms to novel coronavirus (17). Other abnormal lab parameters reported have included coagulation profile abnormalities (18).

In summary, hematologic parameters that are prognostic markers in COVID-19 have not yet been accurately identified in children. Although lymphopenia correlates with increased disease mortality in adults and other biomarkers, including CRP, procalcitonin (PCT), and ferritin, cytokine levels have been associated; these associations have not been reliably tested in children (15). It may be possible that the relative immaturity of the immune system in young children accounts for differences in viral susceptibility or response to infection, possibly explaining the differences in laboratory trends seen in the pediatric versus the adult population of patients with COVID-19 (15). Children with severe COVID-19 showed somewhat consistent trends of elevated lactate dehydrogenase (LDH), CRP, and PCT levels, as reported in adult patients with COVID-19 (16, 20). In addition, elevated D-dimer and PT trends were noted in children with severe COVID-19, although these variables have not been consistently measured across studies (15, 19). Nonetheless, these also overlap with findings reported in adult patients, which have been suggested as markers of the recently highlighted hypercoagulability status seen in patients with severe disease (15, 20). It is worth mentioning that further information on other biomarkers that may help in COVID-19 prognostication, such as IL-6 and serum ferritin levels, remains limited in children, as only two studies reported data on IL-6 in COVID-19, with none reporting data on serum ferritin; IL-6 was only elevated in 37.5% of severe pediatric cases (15). Additional studies should, therefore, incorporate the measurements of IL-6, given that this biomarker is commonly elevated in viral respiratory tract infections and perhaps plays a vital role in the cytokine storm seen with the disease, which may make it part of a risk stratification test [16,22]. Morphologic changes in blood cells have also been reported. Neutrophils show clumped chromatin with toxic granulation and vacuolization, pseudo Pelger–Huet deformity, and bilobed nuclei seen in acute infections (21). Lymphocytes present with abundant pale to dark blue cytoplasm with lymphoplasmacytic features b, and basophilic stippling is seen in red blood cells (21). Platelet clumping has been observed and activated macrophages with abnormal shapes and cytoplasmic vacuolization were also present (21). COVID-19-associated coagulopathy, a distinct form of coagulopathy, has been reported in COVID-19 infection. The pathophysiology involves SARS-CoV-2 entering cells by binding to the angiotensin-converting enzyme 2 receptors; a similar cascade was described earlier. The virus also targets the endothelial cells, which widely express ACE-2 (22). Unchecked viral replication induces a florid host response characterized by dysregulation of inflammation and coagulation. Due to this inflammation and upregulated immune response, platelets are activated, and natural mechanisms of anti-coagulant are downregulated (22, 23). The dysfunction of the endothelium, another risk factor for coagulopathy, may lead to systemic damage with abnormal coagulation, kidney disorders, pulmonary embolism, and sepsis (22, 24). Other pathogenic mechanisms, including increased secretion of von-Willebrand factor (vWF) from damaged endothelium, TLRs, and complement activation, are involved in COVID-19-associated coagulopathy (22). Abnormal hematologic findings related to Covid-19 associated coagulopathy (CAC) include thrombocytopenia, prolonged prothrombin time (PT) and activated partial thromboplastin time (aPTT), and increased fibrin degradation product (FDP) levels, and elevated D-dimer has been associated with poor prognosis and an increased rate of mortality in patients with COVID-19 (22, 25). The most prevalent thrombotic complications of COVID-19 in children parallel those seen in adults and include deep vein thrombosis, pulmonary emboli, chest thrombi, and neurological thrombosis (26). A study reported the incidence of thrombotic events in hospitalized children with COVID-19 as 2.1 and 6.5% in those with MIS-C compared with 0.7% in those with asymptomatic COVID-19 infection (27). Cancer, central venous catheter, older age, and MIS-C are risk factors for thrombosis in children and adolescents with COVID-19 or MIS-C. Mortality was high (28%) in children and adolescents with MIS-C or COVID-19 who developed thrombosis (26). No high-quality evidence demonstrates the safety and efficacy of therapeutic over prophylactic dosing in children. The COVID-19 Anticoagulation in Children Thromboprophylaxis (COVAC-TP) Trial aims to evaluate the safety, dose requirements, and exploratory efficacy of twice-daily subcutaneous enoxaparin as venous thromboembolism (VTE) prophylaxis in children (birth to 18 years) hospitalized with signs and symptoms of SARS-CoV-2 infection (i.e., COVID-19) was launched in June 2020 (27).

Consensus-based clinical recommendations and research priorities for anticoagulant thromboprophylaxis in children hospitalized for COVID-19-related illness (ISTH) have given broad recommendations for AC prophylaxis (28). Among these recommendations include anticoagulant thromboprophylaxis (in combination with mechanical thromboprophylaxis with sequential compression devices where feasible) should be administered in children hospitalized with COVID-19-related illness (e.g., MIS-C) who have superimposed clinical risk factors for hospital-associated VTE or markedly elevated plasma D-dimer levels (e.g., ≥5 times the upper limit of typical values), in the absence of contraindications *[expert opinion, with strong consensus (83%, 15/18)]* (29).

Low molecular weight heparin (LMWH) or unfractionated heparin (UFH) is the anticoagulant of choice in children with acute infection. It is commonly used over UFH due to its reliable pharmacokinetics, pharmacodynamic responses, and longer half-life (28). LMWHs may have additional benefits due to their anti-inflammatory and immunomodulatory properties, i.e., potential antiviral property as it interacts with the SARS-CoV-2 spike S1 protein receptor-binding domain and interferes with its engagement with the receptor (28). Opinions generally differ on monitoring anti-Xa for prophylaxis dosing (28, 29). Higher doses of LMWH/UFH may be needed due to acquired heparin resistance and deficiency of antithrombin as the disease progresses (28).

Bleeding events occur less frequently than thrombotic events and are incompletely understood (18). Microvascular thrombosis and thrombocytopenia are reported to contribute to bleeding events in pediatric patients; cutaneous bleeding, presenting as petechiae, microhemorrhages in the extremities, erythema, macular eruptions, and purpura, often labeled as chilblains was the most common bleeding event reported (18).

In disease-specific hematologic conditions, children with sickle cell infected with COVID-19 were reported to be asymptomatic (25.5%) or to experience mild-to-moderate symptoms (65.6%), while some others had more severe symptoms (8.2%) (30). This cohort was associated with a 40% hospitalization rate, 5.8% ICU admission, 1.1% ventilator use, and 1% death (0.3%) (30, 31). The reported risk factors for worse outcomes included a history of pain and heart/lung comorbidities (30, 31). Our reported sickle cell patients presented with moderate symptoms requiring hospitalization and recovered without additional intensive unit requirements.

Thrombocytopenia is caused by the direct effect of SARS-CoV-2 on platelet production, autoimmune destruction of platelets, or increased platelet consumption (microthrombi formation) (1). A meta-analysis study reported significant thrombocytopenia in patients with more severe than mild diseases (1).

The reported patient with MIS-C developed severe thrombocytopenia, which was deemed multifactorial, and eventually responded to thrombopoietin receptor agonist (TPO-RA), which has since been discontinued due to sustained stable counts. Post-COVID-19 ITP and TTP (potential immune-mediated platelet destruction) have been reported; the likely mechanism is molecular mimicry between the antigens of SARS-CoV-2 and platelet glycoproteins (1). Our young patient developed post-COVID ITP, responded to treatment with IVIG, and continues to do well.

Severe anemia requiring transfusion was not commonly reported in pediatric COVID-19 presentations. However, SARS-CoV-2 could trigger hemolytic anemia (autoimmune) and lead to hemolytic crises in congenital hemolytic anemias (32). Hereditary spherocytosis is inherited hemolytic anemia caused by a genetic mutation that encodes the red cell membrane, resulting in red blood cells having an abnormal, spherical shape with decreased flexibility. Infections/illnesses can exacerbate hemolysis. In a previously reported case, a 4-year-old boy with a history of moderate hereditary spherocytosis (HS) and sickle cell trait without prior splenectomy presented with COVID-19 disease; experienced exacerbated hemolysis requires blood transfusions (33). In our patient vaccinated against COVID-19, hemolysis was mild, and packed red blood cells (PRBCs) transfusion was not needed.

It is important to emphasize that just like any other acute infection/illness, COVID-19 disease can worsen pre-existing or chronic hematologic diseases. For instance, in major patients with thalassemia who are splenectomized, when infected by SARS-CoV-2, these patients may also develop secondary bacterial infections.

As the pandemic continues to wax and wane, efforts need to be intensified to have a comprehensive register explicitly designed for the pediatric population to gather relevant information on the disease presentation, affectation, and clinical course. This will aid the formulation of appropriate treatment guidelines, as well as highlight the areas of focus that need to be improved.

## Data availability statement

The original contributions presented in this study are included in the article/Supplementary material, further inquiries can be directed to the corresponding author.

## Ethics statement

Written informed consent was obtained from the individual(s) and minor(s)’ legal guardian/next of kin for the publication of any potentially identifiable images or data included in this article.

## Author contributions

GO and JA: collection of data, review and reporting of data, and discussion section. AB: review of data and discussion section. All authors listed have made a substantial, direct, and intellectual contribution to the work, and approved it for publication.
