# Supplementary material for: Hematologic manifestations of coronavirus disease 2019 in children: Case-series report and a review
Source: Front Pediatr. 2022 Aug 16;10:935236. doi: 10.3389/fped.2022.935236 (PMC9424539; doi:10.3389/fped.2022.935236)
Supplement: Supplementary file 1 [file Table_1.DOCX]

| **TABLE 1.** Hematological findings in patients with COVID-19 infection   \| **Symptoms** \| **Case 1: MISC with Severe Refractory Thrombocytopenia**  Fever*, multisystem involvement* (respiratory, hematologic, gastrointestinal) \| **Case 2: SCD with VOC pain crises**  Fever, chest, abdominal, and lower extremity pain \| **CASE 3:**  **SCD with VOC pain crises**  Fever, cough, emesis, and lower extremity limp \| **CASE 4:**  **Hereditary Spherocytosis with Acute on Chronic Hemolysis**  cough, congestion, scleral icterus, abdominal pain, nausea, and vomiting \| **CASE 5: Post Covid-19 ITP**  Petechiae, bruising \| \| --- \| --- \| --- \| --- \| --- \| --- \| | | | | | | | | | | | | | | |
| --- | --- | --- | --- | --- | --- | --- | --- | --- | --- | --- | --- | --- | --- | --- | --- | --- | --- | --- | --- | --- |
| **Labs/Days** |  | | | |  | |  | |  | |  | | | |
|  | **Dx** | **P/T** | **D/C** | **F/U** | **Dx** | **D/C** | **Dx** | **F/U** | **Dx** | **F/U** | **Dx** | **P/T** | **D/C** | **F/U** |
| WBC K/uL | 7.7 | 23.7 | 15.3 | 8.4 | 6 | 10.8 | 9.4 | 8 | 9.6 | 8 | 14.4 | 13.4 | 15.1 | 7.3 |
| Neutrophils % | 69 | 73 | 74.2 | 81.1 | 64 | 16 | 32 | 61.9 | 84 | 61.9 | 44 | 47 | 46.7 | 63.7 |
| Lymphocytes % | 10* | 22 | 19.8 | 13.8 | 28 | 65 | 62 | 31.7 | 8.1 | 31.7 | 47 | 46 | 39.5 | 33.2 |
| Monocytes % | 4 | 4 | 4.1 | 4.5 | 6 | 19 | 6 | 4.8 | 7.5 | 4.8 | 6 | 2 | 10.2 | 2.7 |
| Eosinophils % | 0 | 0 | 1 | 0.1 | 1 | 0 | 0 | 1.3 | 0.2 | 1.3 | 3 | 4 | 3 | 0.1 |
| RBC M/uL | 3.3 | 3.19 | 2.91 | 3.38 | 3.26 | 4.51 | 4 | 3.82 | 3.66 | 3.82 | 4.8 | 4.33 | 4.16 | 4.32 |
| Hb mg/dL | 7.8 | 9 | 8.5 | 9.7 | 9.8 | 10.6 | 9.3 | 11.3 | 10.8 | 11.3 | 12 | 11.6 | 10.5 | 11 |
| Platelets K/uL | 83* | 445 | 858 | 844 | 243 | 262 | 142 | 356 | 287 | 356 | 10 | 14 | 40 | 188 |
| Reticulocyte % | - | - | - | - | 3.5 | 3 | 1.8 | 13.7 | 13.6 | 13.7 | - | - | - | - |
| PTT seconds | 32 | 28 | - | - | - | - | - | - | - | - | 44 | 38 | - | 35 |
| PT seconds | 14.6 | 11.8 | - | - | - | - | - | - | - | - | 13.5 | 14.3 | - | 13.4 |
| INR | 1.32 | 1.06 | - | - | - | - | - | - | - | - | 1.22 | 1.29 | - | 1.21 |
| Fibrinogen mg/dL | 624 | - | - | - | - | - | - | - | - | - | 258 | - | - | - |
| D-Dimer ng/mL | 1398* | 576 | - | <200 | - | - | - | - | - | - | 685 | - | - | - |
| Ferritin ng/mL | 350.1* | - | - | - | - | - | - | - | - | - | 37.3 | - | - | - |
| CRP mg/dL | 23.6* | 0.7 | 0.5 | 0.6 | - | - | - | - | 1 | - | <0.5 | - | - | - |
| ESR mm/Hr | 100* | 2 | 6 | 9 | - | - | - | - | <1 | - | 16 | - | - | - |
| **WBC:** white blood cells; **RBC:** red blood cells; **Hb:** hemoglobin; **CRP:** C-reactive protein; **ESR:** erythrocyte sedimentation rate; **PTT:** Partial Thromboplastin Time; **PT:** Prothrombin Time; **INR:** International Normalized Ratio; **Dx:** on diagnosis; **P/T:** post-treatment; **D/C:** on discharge/control; **F/U:** follow up visit; **-:** no data available, **SCD**: sickle cell disease*-**CDC guidelines for the diagnosis of MIS-C**   - *An individual aged <21 years presenting with fever*, laboratory evidence of inflammation**, and evidence of clinically severe illness requiring hospitalization, with multisystem (>2) organ involvement (cardiac, renal, respiratory, hematologic, gastrointestinal, dermatologic, or neurological); AND* - *No plausible alternative diagnoses; AND* - *Positive for current or recent SARS-CoV-2 infection by RT-PCR, serology, or antigen test; or exposure to a suspected or confirmed COVID-19 case within the four weeks before the onset of symptoms.*   **Fever >38.0°C for ≥24 hours, or report of subjective fever lasting ≥24 hours **Including, but not limited to, one or more of the following: an elevated C-reactive protein (CRP), erythrocyte sedimentation rate (ESR), fibrinogen, procalcitonin, d-dimer, ferritin, lactic acid dehydrogenase (LDH), or interleukin 6 (IL-6), elevated neutrophils, reduced lymphocytes, and low albumin* | | | | | | | | | | | | | | |
