# Supplementary material for: Hematologic manifestations of coronavirus disease 2019 in children: Case-series report and a review
Source: Front Pediatr. 2022 Aug 16;10:935236. doi: 10.3389/fped.2022.935236 (PMC9424539; doi:10.3389/fped.2022.935236)
Supplement: Supplementary file 2 [file Table_2.docx]

| **TABLE 2**- Treatment modalities of patients with hematologic disorders and COVID-19 infection | | | | | |
| --- | --- | --- | --- | --- | --- |
| Patients | **Case 1-MISC with Severe Refractory Thrombocytopenia** | **CASE 2**  **SCD with VOC** | **CASE 3**  **SCD with VOC** | **CASE 4**  **Hereditary Spherocytosis**  **With acute on chronic hemolysis** | **CASE 5**  **Post Covid-19 induced thrombocytopenia** |
| Medications | - IVIG (2 doses) - Anakinra - Dexamethasone - Solumedrol - ASA | - Morphine - Toradol | - Morphine Toradol | N/A | - IVIG (1 dose) |
| IVIG 1g/kg/dose Anakinra 2mg/kg and then increased to 6mg/kg, Solumedrol 30mg/kg/day, Toradol 0.6mg/kg/dose Morphine individualized dose per patient | | | | | |
