# Supplementary material for: Hematologic manifestations of coronavirus disease 2019 in children: Case-series report and a review
Source: Front Pediatr. 2022 Aug 16;10:935236. doi: 10.3389/fped.2022.935236 (PMC9424539; doi:10.3389/fped.2022.935236)
Supplement: Supplementary file 3 [file Table_3.DOCX]

| **TABLE 3**- Outcome of patients with Hematologic disorders and COVID-19 infection | | | | | |
| --- | --- | --- | --- | --- | --- |
| Therapy | **Case 1-MISC with Severe Refractory Thrombocytopenia** | **CASE 2**  **SCD with VOC** | **CASE 3**  **SCD with VOC** | **CASE 4**  **Hereditary Spherocytosis**  **With acute on chronic** **hemolysis** | **CASE 5**  **Post Covid-19 induced thrombocytopenia** |
| Outcome following treatment | **R/NPC** | **R** | **R** | **R** | **R/NPC** |
| **R**- recovered from COVID-19 illness. **NPC-** normal platelet counts | | | | | |
